# Editorial: BCIs: research and development in children

**DOI:** 10.3389/fnhum.2023.1201623

**Published:** 2023-05-16

**Authors:** Adam Kirton, Eli Kinney-Lang, James Norton, Tom Chau

**Affiliations:** ^1^Department of Pediatrics, University of Calgary, Calgary, AB, Canada; ^2^National Center for Adaptive Neurotechnologies, Albany, NY, United States; ^3^Department of Biomedical Engineering, University of Toronto, Toronto, ON, Canada

**Keywords:** brain-computer interface, pediatrics, assistive technological devices, communication, severe disabilities

Millions of children living with severe neurological impairment have a limited ability to connect with the world, depriving them of fundamental rights. Brain-computer interfaces (BCIs) are a rapidly evolving potential solution. Despite progress in BCI technology and design and a well-defined need, children have been almost entirely neglected from BCI research. This is changing and this Research Topic provides concrete examples of how fundamental issues are being addressed in the rapidly emerging field of pediatric BCI.

Patient-oriented approaches are critical to the design of BCIs that address the needs of pediatric populations. Keeping with patient-oriented design, Floreani, Rowley et al. identify power mobility as one common goal for families of children living with severe neurological disabilities. They demonstrate the ability of a clinical pediatric BCI program to organize a pilot clinical trial, combining simple, practical BCI systems with established power mobility training techniques. Endorsed by participant families, their results demonstrate the feasibility of such approaches and provide evidence of functional performance that promises to drive future studies.

Hybridization of BCIs with other assistive technologies is a well-considered strategy to maximize performance and practical impact. Although compelling uses for such techniques are easily imagined, research to date for children has been limited. To fill this gap, Mussi and Adams conducted a broad review of hybrid EEG-based BCI systems viewed from the perspective of children with complex needs and their families. Including >40 relevant studies, they describe multiple strategies for the design of hybrid BCIs, including selection of system and paradigm elements, that promise to inform future research efforts and could help bring modern hybridization approaches to pediatric BCI.

The use of BCIs for clinical rehabilitation is a rapidly advancing area of research that has particular relevance to pediatric populations. For example, all the research on BCIs for rehabilitation of stroke-induced hemiparesis has focused on adult populations even though a comparable burden is found in children with hemiparetic cerebral palsy. An initial step toward closing this gap is the first pilot trial of combining BCI with functional electrical stimulation (BCI-FES) in a group of children with perinatal stroke and hemiparesis. Jadavji et al. confirm the ability of children with large brain lesions to operate simple BCI systems and favorable tolerability of pairing motor EEG signals with execution of functional movements in the paretic limb. Phase 2 clinical trials appear to be on the immediate horizon.

Recognizing and liberating the hidden cognitive potential of children with severe physical disabilities is a primary aim of pediatric BCI development. A major challenge is the inability to apply age-appropriate developmental evaluations in children with limited communication abilities. Warschausky et al. describe an important advancement on this issue, evaluating a BCI-adapted version of a standardized test in young people. In demonstrating a strong correlation between the standard and modified versions of the Peabody Picture Vocabulary Test they provide early evidence supporting the psychometric properties of BCI-assisted cognitive assessments. Supplemented by consideration of fundamental challenges of BCI use in children, Huggins et al. further explore specific issues around BCI-based cognitive assessments in an accompanying perspective. Challenges addressed include the design of BCIs for participants with limited alternative communication strategies and practical issues of evaluation design, environmental factors, and interpersonal interactions. These efforts highlight some of the greatest differences between designing BCIs for adult populations, where cognition is often predictably intact, and strategizing BCIs for the more complex brains of children with severe disabilities since birth.

Establishing fundamental design guidelines for BCIs in children will accelerate the adoption of these technologies in pediatric populations. Floreani et al. explored the ability of functional near infrared spectroscopy (fNIRS) to detect emotional valence in typically developing school-aged children. Testing and validating a technique that is well-established in adults like fNIRS-BCI in the developing brain makes fundamental progress toward the use of BCIs by children. Even more innovative and clinically relevant is the concept of capturing and using emotional states. Such paradigms remain unexplored in children despite the high proportion of pediatric BCI candidates who have limitations in communication and cognition where such approaches may be impactful. Their initial validation of the concept merits further investigation while their technique of simultaneously expanding technological and methodological approaches serves as an example for pediatric BCI researchers.

The examples contained here suggest that the unique needs, methods, and opportunities for BCI to impact the lives of children with complex needs are beginning to be defined. Examples are myriad but may include adapting adult approaches to truly unlock BCIs for communication beyond spellers and for those with alternatively developed communication pathways, bidirectional BCI for improving child-centered feedback, how BCIs could be presented and taught for “intuitive” use by children, how current standards of BCI stimuli could be more appealing for kids, how to address attention and fatigue to enable extended use by young users, BCI opportunities for unique pediatric goals such as play, music, art, and social networking, and many others. The number of opportunities for BCI to positively impact the lives of children with severe neurological disabilities would appear unlimited.

## Author contributions

All authors contributed to the editorial and have approved the final version.

